# Supplementary material for: Mutations in PCYT2 disrupt etherlipid biosynthesis and cause a complex hereditary spastic paraplegia
Source: Brain. 2019 Oct 22;142(11):3382–97. doi: 10.1093/brain/awz291 (PMC6821184; doi:10.1093/brain/awz291)
Supplement: awz291_Supplementary_Data [file awz291_supplementary_data.zip › awz291-Suppl_data/Supplementary_Data4.zip!/Supplementary information 3 - fibroblast lipidomics.zip!/Fibroblast lipidomics/Controle vs Patient2/Statistical plots and data/PG species/Boxplots all.pdf]

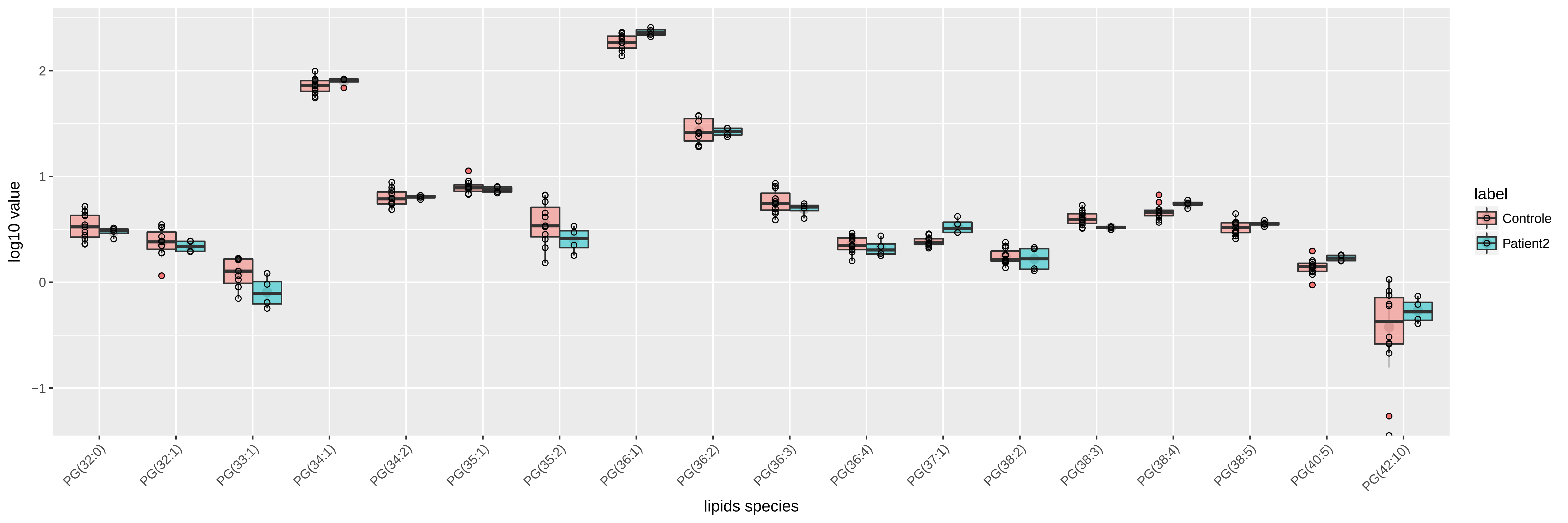

log10 value
2
1
0
−1
PG(32:0)
PG(32:1)
PG(33:1)
PG(34:1)
PG(34:2)
PG(35:1)
PG(35:2)
PG(36:1)
PG(36:2)
PG(36:3)
PG(36:4)
PG(37:1)
PG(38:2)
PG(38:3)
PG(38:4)
PG(38:5)
PG(40:5)
PG(42:10)
lipids species
label
Controle
Patient2
